# Actomyosin-based Self-organization of cell internalization during *C. elegans *gastrulation

**DOI:** 10.1186/1741-7007-10-94

**Published:** 2012-11-30

**Authors:** Christian Pohl, Michael Tiongson, Julia L Moore, Anthony Santella, Zhirong Bao

**Affiliations:** 1 Developmental Biology Program, Sloan-Kettering Institute, 1275 York Avenue, New York, NY, 10065, USA; 2 Buchmann Institute for Molecular Life Sciences, Institute of Biochemistry II, Goethe University, Max-von-Laue-Strasse 15, 60438 Frankfurt, Germany; 3 Program in Computational Biology and Medicine, Cornell University, 1300 York Avenue, New York, NY, 10065, USA

**Keywords:** *C. elegans*, gastrulation, actomyosin, cellular rosette

## Abstract

**Background:**

Gastrulation is a key transition in embryogenesis; it requires self-organized cellular coordination, which has to be both robust to allow efficient development and plastic to provide adaptability. Despite the conservation of gastrulation as a key event in Metazoan embryogenesis, the morphogenetic mechanisms of self-organization (how global order or coordination can arise from local interactions) are poorly understood.

**Results:**

We report a modular structure of cell internalization in *Caenorhabditis elegans *gastrulation that reveals mechanisms of self-organization. Cells that internalize during gastrulation show apical contractile flows, which are correlated with centripetal extensions from surrounding cells. These extensions converge to seal over the internalizing cells in the form of rosettes. This process represents a distinct mode of monolayer remodeling, with gradual extrusion of the internalizing cells and simultaneous tissue closure without an actin purse-string. We further report that this self-organizing module can adapt to severe topological alterations, providing evidence of scalability and plasticity of actomyosin-based patterning. Finally, we show that globally, the surface cell layer undergoes coplanar division to thin out and spread over the internalizing mass, which resembles epiboly.

**Conclusions:**

The combination of coplanar division-based spreading and recurrent local modules for piecemeal internalization constitutes a system-level solution of gradual volume rearrangement under spatial constraint. Our results suggest that the mode of *C. elegans *gastrulation can be unified with the general notions of monolayer remodeling and with distinct cellular mechanisms of actomyosin-based morphogenesis.

## Background

During gastrulation, an embryo is dramatically restructured by cell and tissue movements [1] to position the three germ layers (endoderm, ectoderm, and mesoderm), and to assemble the organ primordia. The paramount morphogenetic task during this process is to internalize surface cells. Four major mechanisms of internalization have been described: invagination (the inward folding of a group of cells), involution (ingrowth and curling inward of a group of cells), ingression (the migration of individual cells from the surface to the interior) and epiboly (growth of a group of cells around another group) [1]. How different organisms bring about this multiplicity of morphogenetic mechanisms that deploy common molecular machineries is poorly understood.

Contractile actomyosin networks are probably the best-studied common molecular assemblies driving morphogenesis [2]. A prominent morphogenetic mechanism that uses pulsatile actomyosin networks during gastrulation is apical constriction [3]. Oscillatory apical contractions of an apical actomyosin network exert pulling forces on discrete cell-cell junctions, which leads to changes in the shape of cells in the tissue [3]. It is thought that these oscillatory contractions cooperatively lead to tissue bending [3,4]. Another example for the deployment of contractile actomyosin networks is epithelial resealing [5]; for example, embryonic wound closure in *Drosophila, Xenopus*, zebrafish, and mouse. In this process,, cells at the epithelial margin form dynamic lamellipodial and filopodial protrusions, and assemble a supracellular actomyosin cable that draws the hole closed, in a similar way to a purse-string.

Both oscillatory contractility and purse-string closures in their canonical form invoke mechanical coherence of individual dynamic components to result in supracellular force-generating systems [6,7]. Interestingly, it has become clear that supracellular structures are very likely not basic structures, but are in fact emergent features of higher organisms [5]. Moreover, contractile behaviors that are apparently similar on the cellular level (for example, oscillatory contractility) can result in markedly different outcomes, depending on the respective subcellular organization and behavior of actomyosin [8].

In the roundworm *Caenorhabditis elegans*, gastrulation begins at the 26-cell stage, when the 2 endodermal cells Ea and Ep internalize on the ventral side of the embryo to form the gut primordium [9,10]. This is followed by the multipolar internalization of mesoderm, primordial germ cells, and ectodermal cells to form the pharynx, body musculature, and neuronal tissue, respectively, all from the ventral side [9,11]. Cell internalization has been mainly studied for the endodermal precursors Ea/Ep, and depends on proper fate specification through Wnt signaling [12-14], on regulators of apicobasal polarity [15-17], on apical accumulation and activation of the protein non-muscle myosin (NMY)-2 [15,18,19], and on cell-cell adhesion [20]. Although apical constriction has been considered a morphogenetic mechanism in light of the requirement of apical NMY-2 for endoderm internalization [10], it has also been shown that mesodermal cells extend over the endoderm, indicating that neighboring cells might actively contribute to internalization [10,21]. How the surrounding tissues and the internalizing cells are coordinated to achieve internalization is not well understood. Notably, cell internalization occurs with a cellular architecture that lacks several aspects of cell-cell coordination compared with, for example, D*rosophila, Xenopus*, or zebrafish; cells have not yet formed coherent tissues, and assembly of polarized apical junctions and deposition of extracellular matrix occur after completion of gastrulation [22,23]. This suggests that clear differences in the cellular mechanisms of gastrulation must exist.

In this study, we investigated the primary morphogenetic module for cell internalization during *C. elegans *gastrulation. During cell internalization, surrounding cells form centripetal extensions that converge into multicellular rosettes to seal over internalizing cells. Extension formation of the surrounding cells correlates with and seems to depend on apical contractile flows in the internalized cells. We showed that this morphogenetic module could adapt to severe topological alterations, providing a mechanistic explanation for plasticity and scalability of gastrulation, which is needed for recurrent deployment of rosette formation throughout gastrulation to internalize different numbers of cells with different sizes and spatial configurations. Besides the recurrent use of this morphogenetic module, we found that globally, coplanar cell divisions thin out and spread the surface cell layer over time. We suggest that the combination of coplanar division and recurrent rosette formation for piecemeal internalization constitutes a system-level solution of volume rearrangement under spatial constraint. Finally, we provide evidence that recurrent rosette formation mediates both local and global rearrangement of cell positions and tissue patterns in the surface layer.

## Results

### Multicellular rosettes seal over the internalizing endoderm

**Figure 1:**
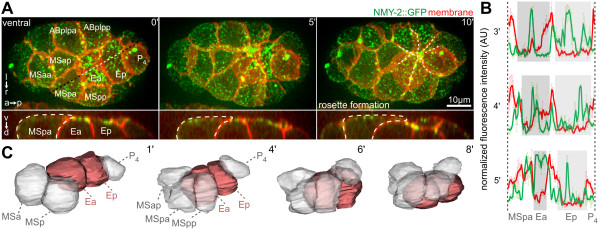
**Rosette formation during endoderm internalization**. **(A) **Dynamics during Ea/Ep internalization. Three-dimensional (3D) projection still images, orientation, and aspect (on the left) and fluorescent markers (in the top right corner) are indicated similarly for each panel throughout the paper. Lower panels depict orthogonal projections along the black dashed line. The final rosette (10 minutes) is highlighted by white dashed lines (see also Additional file 1). **(B) **Correlation between membrane and non-muscle myosin (NMY)-2 dynamics in three representative wild-type embryos. Fluorescence intensities (mean ± SD) were measured on the ventral surface of Ea/Ep along a line similar to the white dashed line in (A). Areas shaded in gray indicate uncovered endodermal cell surfaces. **(C) **3D reconstructions of cells during endoderm internalization. (see also Additional file 2).

To gain insight into the mechanism of cell internalization during *C. elegans *gastrulation, we first investigated endoderm internalization by high-resolution time-lapse microscopy. We found that endoderm internalization was a highly stereotypical process that involved cellular dynamics reproducible to the subminute level. As previously reported, the two endodermal progenitors (Ea/Ep) accumulated contractile foci containing non-muscle myosin NMY-2 on their exposed/apical surface and, simultaneously, three mesodermal (MS) blastomeres extended onto the endoderm (Figure 1A,B; see Additional file 1) [10,21].

Subsequently, other surrounding cells, which include three ectodermal (AB) cells, a muscle precursor (Da) and the primordial germ cell (P_4_), also extended over the endoderm (Figure 1A). This began approximately 7 minutes after the surrounding AB blastomeres had entered and completed mitosis, which occurred 10 minutes after the mesodermal cells started to extend. These centripetal extensions from the surrounding cells converged to form a multicellular rosette that sealed over Ea/Ep (seen in 100% of embryos, n = 13) (Figure 1A, dashed lines; see Additional file 1). While the surrounding cells underwent dramatic shape changes and formed large lateral extensions onto the internalizing cells, the sagittal projections (Figure 1A, lower panel) and 3D reconstructions (Figure 1C; see Additional file 2) showed that the internalizing cells maintained rounded shapes (Figure 1C; see Additional files 2; Additional file 3AB), which is consistent with recent findings that the apical surface of these cells does not accumulate tension during internalization [24]. Moreover, irradiation of surrounding cells led to specific parts of Ea/Ep remaining uncovered (see Additional file 4).

### Recurrent deployment of rosette-based cell internalization

It has been reported that a stereotypical sequence of cell internalization occurs after endoderm internalization [9,11]. These events are all associated with apical accumulation of contractile foci on internalizing cells [10,11]. It has therefore been suggested that cell internalization might rely on a common mechanism.

**Figure 2:**
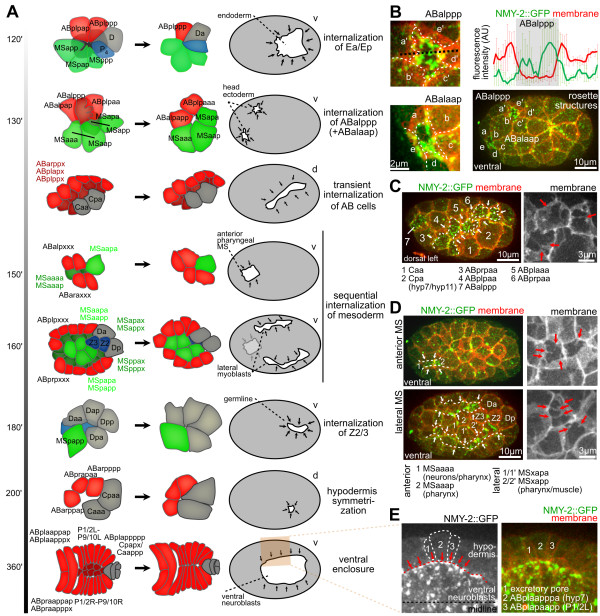
**Later internalizations resemble that of Ea/Ep**. **(A) **Sequence of selected internalization events during gastrulation. Time represents developmental time from fertilization. Schematic representations obtained from imaging data. The area shaded in red during the ventral enclosure is shown in (E). **(B) **Internalization of AB cells in the head region (three-dimensional (3D) projection still images). Intensity-line plots (along the black dashed line in the left panel) for ABalppp were generated as for Figure 1B. **(C) **Dynamics during temporary covering of the lateral AB cell on the left side of the embryo (representations as in (B)); red arrows in the right panel indicated dynamic filopodia-like protrusion from the covering extensions. **(D) **Dynamics during mesoderm internalization. (Upper panel) Internalization of anterior mesoderm; white arrows indicate covering extensions and dashed line mark the leading edge. (Lower panel) Internalization of lateral mesoderm; right panels as in part (C). **(E) **Dynamics during ventral enclosure. Three cells that cover the ventral neuroblasts are labeled for better orientation. The purse-string cable in the covering cells is marked by a dashed red line, and its direction of movement by red arrows.

We found that after gastrulation of Ea/Ep, a rosette-based covering of internalizing cells was repeatedly deployed, consistent with the known order and timing at which cells internalize (Figure 2A-D; see Additional file 1) [9,11]. This also occurred outside the conventional ventral site of gastrulation, , for example, for two ectodermal cells in the head region, ABalaap and ABalppp, which were internalized in 100% of embryos (n = 10) (Figure 2B; see Additional file 1). Additional internalization of ectodermal cells occurred later on the dorsal side (Figure 2C). Notably, the molecular and cellular dynamics of internalization were different from those during ventral closure, where lateral hypodermal cells converged to the ventral midline to cover neuroblasts (Figure 2E). These cells also formed extensions; however, they have also been reported to form an intercellular actomyosin cable that contributes to covering [25], which we did not detect during rosette-based internalization.

Recurrent rosette formation further suggests that both the internalizing and the surrounding cells contribute to gastrulation, and that this mechanism possesses intrinsic structural plasticity that allows internalization of a variable number of cells of different shapes and sizes.

### Plasticity of rosette-based cell internalization

**Figure 3:**
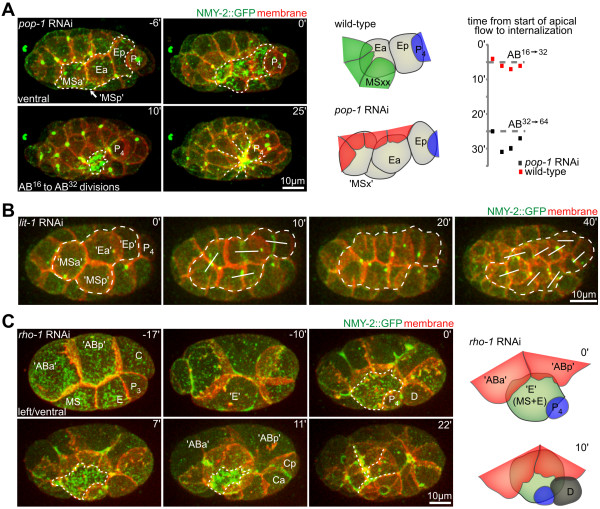
**Plasticity of cell internalization**. **(A) **Ectopic internalization of cells in *pop-1 *RNAi embryos. (Left) Three-dimensional (3D) projection still images: endoderm is outlined with a dashed line, ectopic endodermal cells are labeled 'MSa' and 'MSp'. (Right) Schematic representation of cells involved in covering in wild-type and *pop-1 *RNA interference (RNAi) and timeline of AB extension formation in wild-type and *pop-1 *RNAi embryos (n = 4). **(B) **Lack of contractile flow, extensions and/internalization in *lit-1 *RNAi embryos (representation as in (A)). MS and transformed E cells are outlined with a dashed line. The transformed endoderm did not gastrulate but rather divided on the surface (white lines in panel at 40 minutes). **(C) **Internalization of a fused MS-E hybrid blastomere in a *rho-1 *RNAi embryo (representations as in (A)).

To further investigate the mechanisms underlying rosette formation, we altered the cellular architecture of endoderm internalization genetically in three different ways. First, we generated ectopic endodermal cells using RNA interference (RNAi) with *pop-1*. POP-1 is a TCF transcription factor that acts as a binary fate switch upon the anteroposterior (A-P) divisions [26]. For our purpose, *pop-1 *RNAi transforms mesoderm into endoderm. At the time of endoderm internalization in wild-type embryos, ectopic endoderm in *pop-1 *RNAi embryos accumulated apical NMY-2, similar to endoderm in wild-type embryos (Figure 3A, left panel). Furthermore, cells surrounding the ectopic endoderm formed centripetal extensions and a rosette that sealed over the ectopic endoderm (in 100% of embryos, n = 11). Neither these cells nor their sisters extended over other cells in the wild-type embryos. Thus, ectopic extensions are not due to A-P fate transformations of the surrounding cells, but are induced by the ectopically internalizing cells.

Second, in the converse experiment, we used *lit-1 *RNAi to transform Ea/Ep into mesoderm [27]. LIT-1 is a Nemo-like kinase that negatively regulates POP-1. In *lit-1 *RNAi embryos, Ea/Ep lost the higher level of apical NMY-2, and the surrounding cells did not form centripetal extensions (Figure 3B). These findings are supported by previous studies using a temperature-sensitive mutant of *lit-1*, which showed that *lit-1 *is not required for internalization *per se *[10]. Taken together, these results suggest that proper specification of the internalizing cells is required to activate rosette formation and internalization.

Third, reduction of the small GTPase RHO-1 by RNAi generated partially syncytial embryos. Specifically, although the embryonic axes formed normally and the first two rounds of divisions seemed to be normal, subsequent cleavage furrows were able to reopen and cells fused (Figure 3C) [28]. Remarkably, when the mesodermal founder cell MS and the endodermal founder cell E fused, the hybrid MS-E cell exhibited the behavior of Ea/Ep, with apical accumulation of NMY-2 and internalization timing comparable with wild-type cells, and with the cell covered by surrounding extensions.

These perturbations confirm the structural plasticity and robust formation of rosettes, with a wide range of spatial configurations of internalizing and surrounding cells. More importantly, these experiments suggest the following model of self-organized rosette formation. Although most cells in the embryo seem capable of extending over and covering neighboring cells, fate-specification pathways determine which cells will become internalized, and thus, this cellular mechanism provides the modularity and structural plasticity of rosette formation.

### Correlation of apical contractile flows in internalizing cells and rosette formation

Developmentally regulated apical contractile flow is an important mechanism to mediate gastrulation and cell internalization [3,24,29]. Given the apical accumulation of NMY-2 in the internalizing cells, we investigated whether apical contractile flows correlate with extension and rosette formation of the surrounding cells.

We found that the apical dynamics of NMY-2 in Ea/Ep showed two distinct phases. In the first phase, the dynamics of NMY-2 in Ea/Ep were similar to those in other cells after they are born. Small NMY-2 foci (marked with green fluorescent protein; GFP) emerged at the cell periphery, flowed towards the apical center, and disappeared before they could cluster at the apical center (see Additional file 3, panels C,D). Concomitant with these apical NMY-2 flows, PAR-6 accumulated on the apices of Ea/Ep (see Additional file 3, panel F) [10,30]. Subsequently, the apical PAR-6 domain in Ea/Ep shrank as a result of extension advancement, while the basolateral PAR-2 domain expanded and eventually completely covered the Ea/Ep surfaces (see Additional file 3, panel G). This indicates that the cell contact-mediated specification of the PAR-6 and PAR-2 domains, which defines the inside-outside polarity in the surface cell layer [17], remodeled the polarity in the internalizing cells during rosette formation.

**Figure 4:**
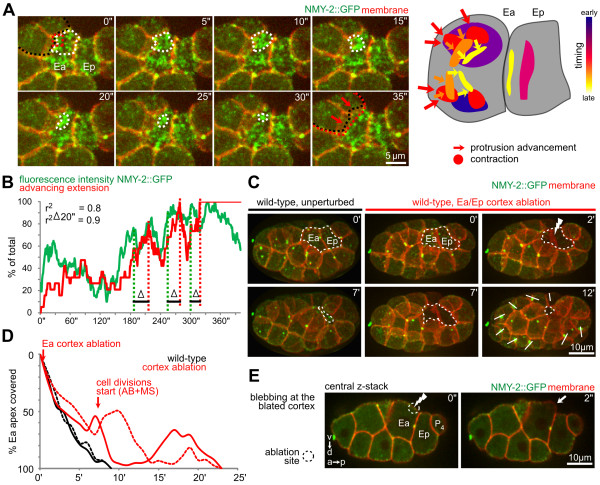
**Relationship between apical contractions and extension advancement**. **(A)**(Left) Spatiotemporal correlation of contractions and extension advancement in a representative embryo. Contractions are outlined with a dashed white line, while initial and final position of neighboring cell extensions are labeled with a black and red dashed line, respectively. Red arrows indicate the direction of extension advancement. (Right) Schematic representation of multiple contractions in a representative embryo. Colored areas represent initial spread of contractile foci that cluster into one focus, and arrows indicate contractions coupled to extension advancement; the order of contractions is color-coded. **(B) **Graphs correlating integrated local fluorescence intensity of non-muscle myosin (NMY)-2 labeled with green fluorescent protein (NMY-2::GFP) with advancement of an adjacent extension. Fluorescence was normalized to maximal integrated intensity, and extension advancement was normalized to the final position of the tip of the extension in the rosette. **(C) **Apical contractile flows were required for rosette formation. The panels show a comparison of endoderm covering in (left) an unperturbed embryo and (middle) an embryo in which the apical cortex of Ea and Ep was ablated (lightning) by laser. These panels show three-dimensional (3D) projection still images, and uncovered endoderm is highlighted by a dashed line. **(D) **Quantification of covering of endoderm in two wild-type embryos and in two embryos in which the endodermal cortices were ablated by laser. **(E) **A large bleb formed at the ablated cortex. Two-dimensional (2D) confocal slices at higher temporal resolution reveal the site of ablation (dashed circle/lightning), cortex rupture (arrow), and extensions (open arrows).

In the second phase, the NMY-2 foci clustered and contracted, and these contractions were followed by extension advancement of surrounding cells. Specifically, about 18 minutes after Ea/Ep were born, and around the time the neighboring mesodermal cells exited mitosis, the number of NMY-2 foci markedly increased, and streams of NMY-2 foci converged and contracted (Figure 4A). During internalization, the endoderm usually exhibited more than 10 apical contractions (Figure 4A, right panel). Contractions adjacent to cell-cell contacts were accompanied by advancing extensions of the surrounding cells (Figure 4A, arrows in the 35-second frame). When a local contraction relaxed, the trailing extension partially retreated, and when a new contraction occurred nearby, the extension advanced again in the direction of this contraction. Quantitatively, extension advancement correlated well with local contractions with a delay of approximately 20 seconds (Figure 4B, r^2 ^= 0.9 with a 20-second time shift). The time-shifted correlation suggests that contractions may be coupled to extension advancement, with the delay being consistent with the Ea/Ep apical cortex being viscoelastic [24,31].

To test whether apical contractions are required for extension advancement, we performed laser-ablation experiments, which showed that apical contractions were indeed required for extension advancement. In previous experiments, a finely focused UV laser was used to cut the cortex without abolishing contractile foci [24,31], whereas in the current study, we ablated the entire apical cortex in Ea and Ep. We ensured that the cortex was effectively disrupted by detecting formation of a large bleb at the ablation site (Figure 4E, right panel) [32] and a lack of fluorescence recovery across the cortex. We found that ablations of the Ea/Ep cortex abolished extension advancement and rosette formation (Figure 4C,D).

### Apical contractions and extension formation independent of gastrulation movements

We performed a more detailed analysis of apical contractions to investigate whether they might have a more general role. First, we found that ectodermal cells known to stay on the surface of the embryo; for example, ABplapp, which mainly generates the hypodermis) showed apical contractions that were qualitatively similar to but less intense than those in Ea/Ep. These flows were followed by small extensions from neighboring cells and partial covering (see Additional file 3E, upper panel). However, before cell division occurred, apical clusters of NMY-2 dissolved. Consistently, the extensions of surrounding cells were reproducibly retracted and cells stayed on the surface (see Additional file 3, panel E, bottom, n = 3). This observation indicates that cells could establish stochastic apical contractions that were below the threshold at which extensions can be maintained. It also further strengthens the notion that cells were generally competent to extend and cover other cells.

**Figure 5:**
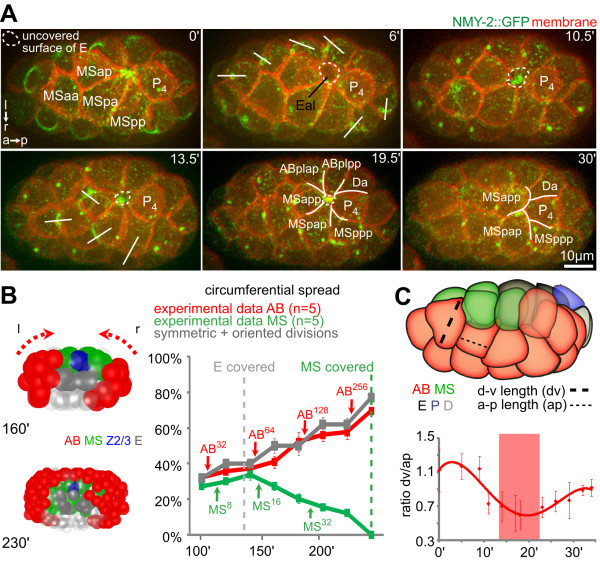
**Coplanar cell divisions during gastrulation**. **(A) **Coplanar divisions during Ea/Ep internalization. Time-lapse microscopy with three-dimensional (3D) projection still images. Cytokinetic rings in AB and MS cells are present in frames at 0 and 13.5 minutes, while the white lines at 6 and 13.5 minutes indicate division directions. Note that when AB cells divided and retracted their extensions (frame at 6 minutes), NMY-2 gradually reappeared at the resurfacing part of the endoderm (between frames at 6 and 10.5 minutes). **(B) **Dynamics of circumferential tissue spreading. (Left) Posterior of a lineaged embryo; circumferential movements are indicated by red dashed arrows. (Right) Quantitative analysis of circumferential tissue movements and comparison to theory; graphs indicate the degree of circumferential spread (mean ± SD). **(C) **Morphometric analysis of cell-shape changes in the surface layer during Ea/Ep internalization. The graph depicts the change in the ratio between apical and basolateral axis length for three surface cells (ABaraa, ABarap and ABpraa) that were not involved in rosette formation (n = 3, mean ± SD, polynomial fit).

Second, when the integrity of the surface layer was interrupted by cell division, the re-exposed apical surfaces of cells again exhibited contractions followed by rosette formation and resealing (Figure 5A). This indicates that even after internalization, cells maintain' a morphogenetic program to re-establish apical contractions when their apical surfaces become exposed again. Because accumulation of NMY-2 and apical contractions required the correct cell fate (Figure 3B) [10], this mechanism is very likely linked to cell fate maintenance, which provides robustness to the gastrulation process.

Third, the apical surface of Ea/Ep blebbed during internalization (100% of embryos, n>10) (see Additional file 5A). The size and dynamics of these blebs were comparable with those of blebs in other systems that were caused by cortical contractions (see Additional file 5, panel A, lower right) [33,34]. Blebs were rapidly retracted, typically within 30 to 60 seconds, which presumably allowed further contractions to exert forces on lateral extensions. The endoderm usually showed more than 10 contractions during internalization, but only some of these led to extension advancement (Figure 4B). As bleb formation can dissipate contractile energy [35], blebbing and the underlying rupturing may explain these unproductive contractions. Consistent with blebbing being caused by contractions, weak RNAi of *nmy-2 *reduced both contractions and blebbing (see Additional file 5B).

### A local mechanism for global cell organization in the embryo

The pre-gastrulating 26-cell embryo is essentially an ellipsoidal monolayer, with all cells being on the surface and no free space inside except for a microscopic blastocoel [10]. Combined with the confinement of the eggshell, this configuration poses a volumetric constraint for gastrulation. Our observations suggest that first, rosette formation allowed gradual rearrangement of volume and surface area as cells internalized and the embryo was transformed into a two-layered and subsequently three-layered structure. Second, resolution of rosettes was accompanied by synchronized cell divisions polarized in the plane of the surface monolayer that encompassed the entire embryo (Figure 5A: NMY-2::GFP showing orientation of contractile rings and cell divisions in frames at 0, 6 and 13.5 minutes) [36]. The coplanar divisions dictate that the monolayer will be thinner and occupy more surface area. Thinning can spare inner space to accommodate internalizing cells and the spreading can relieve tissue tension in the rosette and finalize covering. Indeed, in our study, ectodermal cells on the surface layer continued to undergo planar polarized divisions throughout gastrulation (Figure 5B, left panels; see Additional file 1). The measured circumferential spreading of the ectoderm can be perfectly explained by theoretical calculation of coplanar divisions based on the known average spindle orientation, which is 36° from the A-P axis because of spindle rotations (Figure 5B, right panels) [36].

This notion is supported by experimental perturbations. As explained above, *pop-1 *RNAi generated two ectopic endodermal cells, doubling both the volume to be internalized and the surface area to be covered by the rosette. As expected, it took an additional division of the surface cells for the extensions to form a rosette and another division to resolve the rosette and finalize the internalization of the enlarged endoderm (Figure 3A, 25-minute frame in left panel; n = 4 for both wild-type and *pop-1 *RNAi embryos in right panel). Interestingly, analysis of ectodermal (AB) cell shapes during endoderm internalization revealed a general transition of the surface cells, from cuboidal to columnar upon division, and back to cuboidal after the spreading (Figure 5C). Acquisition of these columnar shapes suggests that coplanar divisions cause cell-shape deformations through temporary crowding in the plane of the monolayer, the pressure of which could facilitate endoderm internalization. Moreover, when transformed cells that would otherwise internalize in wild-type embryos stayed on the surface of in *lit-1 *RNAi embryos, cell crowding on the surface of the embryo could be seen and other cells internalized instead (data not shown).

Taken together, our analyses suggest that coplanar division combined with rosette formation provides a systems-level permissive mechanism for gastrulation involving the surrounding and other cells in the surface layer.

### Roles of surrounding cells in rosette formation

**Figure 6:**
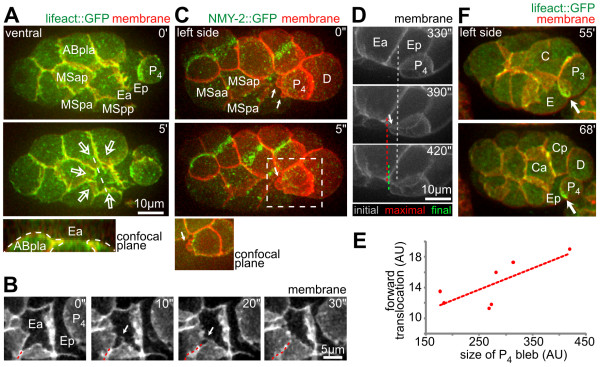
**Contribution of cell-specific mechanisms to rosette formation**. **(A) **Dynamics of F-actin (lifeact) localization during endoderm internalization. (Top left) Three-dimensional (3D) projection still images, ventral views; arrows indicate the direction of covering. (Bottom left) The x-z projection along the dashed white line (frame at 5 minutes). Note that actin-rich structures on Ea were part of the covering cells (outlined with thin dashed lines). **(B) **Ventral views onto Ea/Ep; arrows indicate a filopodium that established a lateral contact (dashed line) between neighboring extensions, followed by sealing. **(C) **Blebbing in P_4_; arrows point to blebs formed at the anterior side. The boxed area (5 seconds) is shown enlarged on the bottom of the frame as a two-dimensional (2D) confocal image to illustrate separation of plasma membrane and cortex. **(D) **Blebbing led to anterior translocation. A blebbing event during late stages of Ep covering is shown, with 2D confocal side views, initial anterior position of P_4_, maximal position after blebbing, and final position after repair of the cortex. **(E) **Correlation of cross-sectional bleb area and anterior translocation of P_4 _from three wild-type embryos. A linear regression is shown as a dashed line. **(F) **Cellular history of the P_4 _'actin brush' (representations as in (A)). White arrows mark the actin brush. Time is developmental time from fertilization.

Our observations further indicate local roles of surrounding cells in rosettes. First, the centripetal extensions showed polarized localization of NMY-2 and F-actin at their tips (Figure 1B, green curve in the panels at 4 and 5 minutes panels in MSpa; Figure 6A). More interestingly, we found dynamic filopodia at the tips of adjacent extensions (Figure 6B), and we speculate that these filopodia might coordinate lateral contacts between extensions and thereby result in complete sealing during rosette formation.

Second, closer examination showed that P_4_, the germline precursor, behaved differently compared with the other surrounding cells during Ea/Ep internalization and showed directional motility similar to zebrafish primordial germ cells [37]. Specifically, P_4 _formed blebs towards the gastrulation cleft, driven by stochastic rupture of the actomyosin cortex (Figure 6C). We found that each blebbing event led to anterior translocation of P_4 _and that the size of a bleb correlated with the distance of anterior translocation (Figure 6D,E). Furthermore, P_4 _polarized F-actin along its basal side, contacting Ep at the leading edge in structures (Figure 6F) that have been described in zebrafish as 'brushes' [37]. This polarization also occurred between the corresponding grandmother cells two cell cycles earlier (Figure 6F, 55 minutes) [38], and was maintained until completion of Ep internalization. Together, these results suggest that directional blebbing might constitute a more widespread mechanism for the motility of primordial germ cells.

### Rosette formation and monolayer remodeling

The stereotypical formation and resolution of the rosette covering Ea/Ep generated new cell-cell contacts and adjusted relative cell positions within the surface layer (Figure 5A; see Additional file 1). From the joint point contact of the rosette, invariant rosette resolution led to contact formation between the primordial germ cell P_4 _and the mesoderm (compare Figure 5A (30 minutes) with Figure 1A (0 minutes)). This in turn allowed the mesoderm to cover the primordial germ cells, which brought into juxtaposition the muscle sublineages derived from the MS and D blastomeres, so that the anterior and posterior halves of the musculature became contiguous (Figure 5A, 30 minutes; see Additional file 1). Notably, when cells in this rosette divided, they behaved in accordance with Hertwig's rule [39,40]: the spindle 'lies in the longest axis of the protoplasmic mass, and division therefore tends to cut this axis transversely' (see Additional file 5C). With the spindle oriented along the extensions as seen in the current study, the local tissue topology generated during rosette formation effectively regulates the local remodeling of relative cell positions in the surface layer throughout gastrulation.

**Figure 7:**
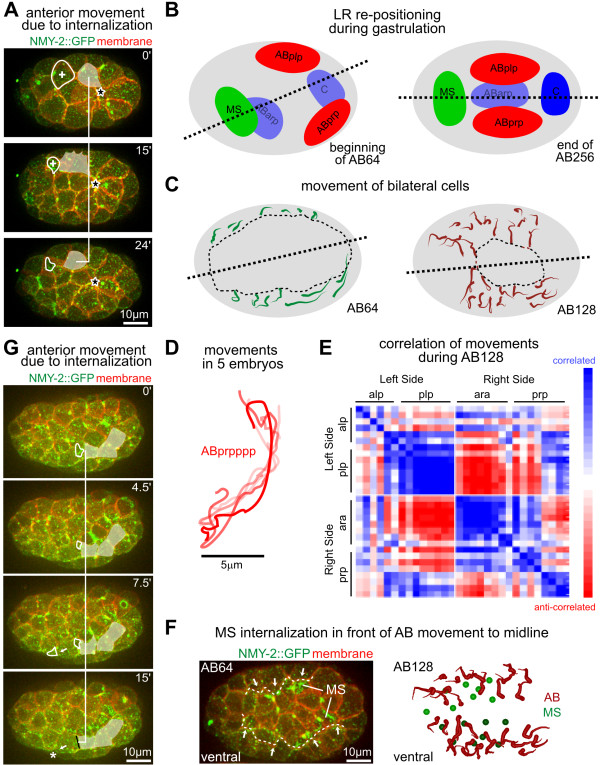
**Internalization events organize local and global tissue movements**. **(A) **Three-dimensional (3D) projection stills showing movement of ABplpap while covering ABalppp. White shade marks ABplpap, while white circle and plus (+) sign mark ABalppp and its apical contractile flows during internalization. Black star marks the rosette covering Ea/Ep, in which ABplpap participated initially but left. **(B) **Schematic representation of lineage groups before and after the establishment of superficial left/right symmetry. Dashed lines represent the midline. **(C) **Movement paths of bilateral AB cells (ABalp, ABara, ABplp, ABprp) at AB64 and AB128). Th path of each cell was averaged over five lineaged embryos, and the thin end marks the start position. The paths of the sister cells have beencombinedinto a single path for visual clarity. Dashed lines represent the midline, which moved toward the anterior-posterior (A-P; long) axis of the embryo as cells moved. Dashed circles mark the remaining gastrulation cleft. **(D) **Individual migration paths for the ABprppp cell from five lineaged embryos. **(E) **Correlation analysis of movements; the matrix heat map representation shows correlation of paths of individual AB cells (same cells as in (C)) at the AB128 stage along the left/right axis. **(F) **Bilateral AB cells moved towards the midline as they covered MS cells. (Left) Three-dimensional (3D) projection image at AB64. The dashed line marks the leading edge of the AB cells, and arrows show direction of movement. MS cells can be recognized by their apical contractile flows. (Right) Schematic representation of continued AB movement in the next cell cycle to finish covering the MS cells. **(G) **Anterior movement of AB cells on the right side of the embryo (ABprp) upon two internalizations in front of the movement. White shade marks three AB cells, and white circles mark internalizing cells. The black line in the final frame marks the complete sealing over of an internalizing cell, while star marks the other internalization.

Rosette formation is also associated with cell movements. For example, ABplpap was born while engaged in the rosette covering Ea/Ep (Figure 7A). In some embryos, it was then recruited into the rosette covering another cell (ABalppp) on the anterior side. During this process, ABplpap moved anteriorly for about 11% of the embryo length. In other embryos, the sister of ABplpap was recruited to the anterior rosette, as a result of slight variation in cell positions (see Additional file 1). In this case, the anterior displacement of ABplpap during rosette formation was less than 5% of embryo length. This contrast suggests that rosette formation can mediate significant movement of covering cells.

### Coordination of gastrulation and acquisition of superficial left-right symmetry

Systematic quantification of cell positions over time showed significant global cell movements during gastrulation [41], and the final positions can be explained by cells sorting among themselves, based on comparison of A-P identity between neighbors [42,43]. We found that one theme of the global cell movement was the adjustment of the midline and acquisition of superficial left/right symmetry (Figure 7B), as we reported recently [44]. The midline is established during chiral symmetry, breaking at the six-cell to eight-cell transition, which tilts to the right from the A-P axis [44]. The resulting asymmetric cell positions allow a sequence of differential Notch signaling to break bilateral fate symmetry [45]. The tilted midplane is then aligned with the A-P axis through cell movements to establish superficial left/right symmetry [44].

Gastrulation and the acquisition of superficial left/right symmetry were tightly coordinated during development. The overall movements during this period can be decomposed into two mechanism. First, bilateral groups in the ectoderm, such as descendants of the ABplp and ABprp cells, moved towards the midline to close the ventral gastrulation cleft (Figure 7C,D). This was reflected by the correlation of movement paths within each side and the anti-correlation across the two sides (Figure 7E). Second, the ABprp descendants on the right side, which were initially located more posteriorly than their left counterpart ABplp, moved anteriorly, so that the midline moved towards the A-P axis (Figure 7C,D). The size and orientation of the remaining gastrulation cleft were correlated (Figure 7C), so that by the time the cleft was closed at the 350-cell stage, the midline and the A-P axis coincided [44].

We further found that the position of internalizing cells correlated well with both mechanisms of the movement described above. The covering cells moved towards the space occupied by internalizing cells (Figure 7F,G; see Additional file 1). In particular, we found that two internalization events anterior to the ABprp cells were accompanied by the anterior movement of these cells, which was about 5% of the embryo length (Figure 7G). However, the movement in this time window, which is about half of the cell cycle, accounted for only half of the anterior movement of these cells (Figure 7D, n = 5 embryos). Therefore, other mechanisms such as active migration [42,43] must be needed for the adjustment of the midline. Nonetheless, these results, together with the analysis of rosette formation and cell movement at early stages (Figure 7A), suggest that rosette formation not only mediates cell internalization, but also contributes to the organization of tissue movements in the surface layer, including those that generate superficial bilateral symmetry.

## Discussion

### A local morphogenetic module

Our results reveal a uniform mechanism of cell internalization in *C. elegans *gastrulation that is modular and scalable (Figure 3). Surrounding cells formed centripetal extensions that converged into a multicellular rosette to seal over internalizing cells (Figure 1). This mechanism was deployed repeatedly to internalize small numbers of cells at different locations throughout gastrulation (Figure 2). Furthermore, our results suggest that the internalizing and surrounding cells form a morphogenetic module, so that while the internalizing cells exhibited apical contractions, the surrounding cells formed centripetal extensions. Recent studies have shown that adhesion is required for *C. elegans *gastrulation [20] and that reduction of the E-cadherin HMR-1 prevents extension formation without affecting contractile flows [24], indicating that cell-cell adhesion is probably responsible for transducing the force from apical contractions to the surrounding extensions. A recent study using biophysical measurements of embryonic zebrafish cells also demonstrated that adhesion through E-cadherin is needed to mechanically couple the cortices of adhering cells at their contacts, allowing cortical tension to control shape changes [46]. We therefore suggest that apical contractions and polarized extensions constitute a module through mechanical coupling between cells. Apical contractions close to cell-cell contacts led to subsequent extension advancement (Figure 4A), whereas apical contractions further away from cell-cell contacts led to blebbing and thus dissipation of contractile force (see Additional file 5A). Notably, our data using time-lapse microscopy provides only correlative evidence for mechanical coupling; we currently do not have experimental evidence for a signaling event that couples contractions to extension formation and would thus favor a simpler model of mechanical coupling.

Using genetic perturbations, we found that the morphogenetic module of rosette formation could be altered dramatically by RNAi. Interestingly, these extreme phenotypes resembled normal development in distantly related nematodes (Figure 3; see Additional file 5D)., suggesting that the inherent plasticity and robustness of the rosette module may have been exploited over evolution [47,48]. Moreover, our experiments also indicate that this module relies solely on cells having acquired the correct fate that allows them to activate apical contractility and thereby their own internalization (Figure 3). For a biological system, this can be considered self-organization, in the sense that the rules specifying cell-cell interactions are executed based on local information, without reference to the global pattern [49]. In addition, these experiments also revealed that this morphogenetic module is very robust; for example, as long as the endoderm is specified (independent of the number of cells) internalization will take place. Moreover, the fusion of mesoderm and endoderm in *rho-1 *RNAi shows that endodermal fate seems to be able to 'overwrite' the fate of the mesoderm.

### Global organization of gastrulation by local mechanisms

Our analysis also demonstrated a second level of self-organization, namely that interactions of a few components of the system affect global level behavior [49]. First, during gastrulation, the surface layer underwent synchronous and coplanar cell divisions to thin and spread over the internalizing mass (Figure 5), and it has been shown that a Wnt-dependent local relay mechanism is responsible for maintaining this polarization during gastrulation [36]. These coplanar cell divisions may further enrich our understanding of how a field of cells acquires a common sense of direction in addition to the canonical planar cell polarity (PCP) and the recently proposed planar cell chirality [50]. It has been suggested that MOM-5/Frizzled, which regulates spindle orientation, behaves more like the PCP pathway than the canonical Wnt pathway, and that moreover, the asymmetric distribution of signaling pathway components does not depend on extrinsic polarity cues [51]. Taken together, these findings all support a mechanism of global patterning (coplanar divisions) based on local interactions (a Wnt/PCP-like pathway) [42].

Second, internalization events were accompanied by the movement of covering cells towards the space occupied by the internalizing cells (Figure 7). Thus, it is conceivable that rosette formation functions as a local morphogenetic organizer, and that positioning of internalizing cells orchestrates a network of cell movements that together mediate global tissue remodeling (Figure 7B). Importantly, our data support that at least one organism-level property seems to involve this mechanism, namely, the acquisition of superficial bilateral symmetry during late stages of gastrulation. However, as our measurements show, additional mechanisms such as active migration are also involved. It remains to be elucidated how different movement mechanisms contribute to the cell sorting based on local comparison of cell identity between neighbors [42,43,46], and how these mechanisms integrate to achieve global pattern formation.

### Comparison of morphogenetic mechanisms

Our results reveal rosette formation as the primary mode of cell internalization during *C. elegans *gastrulation. Rosette formation is only a kinematic description of cell-shape changes that reflect a common geometric constraint but not necessarily common cellular mechanisms; however, our analyses suggest that rosette formation in *C. elegans *bears common features with known mechanisms and concepts of morphogenesis at the molecular and cellular levels as well as on the whole-embryo level.

Rosette formation has been best described in the *Drosophila *embryo during axis elongation [52]. In this process, multicellular rosette structures are formed by intercalating populations, in which five or more cells meet at a single point [53]. These rosettes resolve in a directional fashion, elongating the cellular array along the A-P axis and acting as an amplifier of neighbor exchange. Despite the similarity in cellular topology, the underlying molecular dynamics in the two organisms are different. Rosette formation in the *Drosophila *embryo requires an alignment of actomyosin structures across multiple cells to form cables along cell contact that contract together [54]. The cellular mechanism of *C. elegans *gastrulation seems in fact to be more similar to dorsal closure in *Drosophila *[55], with internalizing cells undergoing oscillatory apical contractions and a substantial fraction of cells internalizing near the leading edges of lateral epidermis, consistent with our findings that cell internalization also depends on surrounding cells. However, a key difference from *Drosophila *is that the leading edges in *C. elegans *are again not connected by supracellular actin cables. The *C. elegans *embryo is capable of forming such cables during ventral enclosure, which occurs during later development [25]. Interestingly, the use of supracellular actin cables in *C. elegans *is associated with the degree of epithelial polarization; the covering cells in the ventral enclosure form apically polarized cell-cell junctions, whereas in earlier cells, the junctional proteins are distributed more widely on the basal side [22]. It is not clear what are the advantages to having two different mechanisms (rosette-based covering or purse-string actin cables, respectively) for early and late embryogenesis, or what controls the developmental transition. We can only speculate that in order to achieve coherent movement of large numbers of cells, supracellular structures facilitate synchronous morphogenetic movements more robustly.

Our study suggests that at the embryonic level, *C. elegans *gastrulation can be viewed as monolayer remodeling, as in many other organisms. In this light, the surface layer can be considered as a primitive or specialized form of epithelium, with apical localization of PAR (abnormal embryonic PARtitioning of cytoplasm) proteins [10], adhesion, and junctional proteins [20,22,24], along with planar polarity (see above). The surface layer thins and spreads over the internalizing mass as in gastrulation of other organisms. Rosette formation mediates the exclusion of cells from the epithelial sheet while maintaining continuity of the sheet, and also mediates both local and global restructuring of cell positions within the sheet. Although some of the features are less pronounced as a result of the spatial constraint of the eggshell, these interpretations unify the gastrulation of *C. elegans *with the general framework.

## Conclusions

We have shown that multicellular rosette-based covering is a major morphogenetic module of gastrulation in *C. elegans *that confers structural plasticity and scalability. Although direct experimental evidence is lacking, it seems plausible that this module functions by coupling of apical contractions in internalizing cells to extension formation of surrounding cells through mechanical links between these cells and their cortices [24,46]. We also propose that internalization through this mechanism represents an example of self-organization by which local morphogenetic processes can function as organizers of global tissue movements within the surface layer of the embryo, and contribute to the establishment of superficial bilateral symmetry.

## Methods

### *Caenorhabditis elegans *strains and handling

Worms were cultured at 20 to 25°C using standard procedures [56] and were well fed for at least two generations before embryos were collected from young adults and imaged at 20° on a temperature-controlled stage. Strains used in this study were: OD70 (unc-119(ed3)III; ltIs44pAA173; [pie-1::-mCherry::PH(PLC1d1) + unc-119(+)]V); JJ1473 (unc-119(ed3)III; zuIs45[nmy-2::NMY-2::GFP + unc-119(+)] V); RW10049 (unc-199 (ed3)III; zuIs178 [(his-72 1kb::HIS-72::GFP) + unc-119(+)]; stIs10050[pha-4(4kb)::HIS-24::mCherry + unc-119(+)]); RW10226 (unc-119(ed3) III; itIs37[pie-1::mCherry::H2B-pie-1UTR + unc-119(+)]; stIs10226[his-72 promoter::HIS-24::mCherry translational fusion with let-858 3' untranslated region (UTR) + unc-119(+)]); and FT17 (xnIs3[par-6:PAR-6::GFP + unc-119(+)]). Strains containing several markers were obtained by crossing these parental strains. The generation of lifeact-expressing strains has been described recently [44]. Several strains were obtained from the Caenorhabditis Genetics Center (CGC) at the University of Minnesota, Twin Cities.

### RNA interference treatment, embryo preparation, imaging, and image analysis

Embryo treatment and handling were carried out as described previously [44]. Briefly, embryos were dissected from gravid hermaphrodites, mounted in 2.5 μl of an M9 buffer suspension containing 25 μm polystyrene microspheres (Polyscience, Warrington, PA, USA), and sealed between two coverslips (Corning, Lowell, MA, USA) with petroleum jelly. Images were acquired using a spinning disc confocal system (Wave FX; Quorum Technologies, Guelph, Canada) and microscope (Observer Z1 microscope; Carl Zeiss Microimaging, Jena, Germany) with oil-immersion objectives (PlanApo 40x/1.3 or 63x/1.4 Oil objectives; Carl Zeiss Microimaging). Dual-color images were acquired using two simultaneously operating digital recording cameras (EM-CCD; Hamamatsu, Hamamatsu City, Japan) usually in the streaming mode of the acquisition software used (MetaMorph; Molecular Devices, Downington, PA, USA) to reduce recording time. The intensities of the diode-pumped solid-state (DPSS) lasers (Calypso (491 nm), Jive (563 nm) and Mambo (591 nm); all Cobolt AB, Solna, Sweden) were adjusted using the MetaMorph software and an acousto-optical tunable filter.

Unless indicated otherwise, data are represented as maximum-intensity projections of a *z*-stack of 20 to 30 sections at 0.7-1 μm spacing, generated with MetaMorph software or with custom plug-ins to ImageJ software (http://rsbweb.nih.gov/ij/). When images were rotated, linear interpolation was applied. Time-lapse animations were generated using a custom MATLAB (The Mathworks Inc., Natick, MA, USA) routine and ImageJ software. Movies were normally generated with JPEG compression. Kymographs (see Additional file 3, panel D) were generated from *z*-stacks with cortical signals only. Reprojections in x-z of 2D data in x-y were performed using the Image>Stacks>Reslice function in ImageJ. To achieve a clearer representation, a 0.5 pixel Gaussian blur filter was applied to reprojections.

### Measurements from fluorescent time-lapse images and AceTree data

Histograms of fluorescence intensity were generated from ventral *z-*stacks, using the 'Plot Profile' tool in ImageJ. The graphs display fluorescence intensity as measured on two lines spaced at 1 pixel parallel to and on the line indicated in the images, and data are shown as mean ± SD. Rosette structures were manually segmented. Measurements of the circumferential spread were performed from AceTree (http://acetree.sourceforge.net) data [57-59]. AceTree images displaying a cross-sectional view from posterior at the midpoint of the embryo were imported into ImageJ, and the circumferential spread was calculated as percentage of the circumferential expansion of the respective tissue relative to the whole circumference of the embryo. To obtain the total circumferential spread of ectoderm (AB), the two lateral halves were added. To calculate the theoretical circumferential spread, cell-shape changes during cell division were assumed to be perfectly symmetric. Division timing was taken from the measurements from real embryos. Furthermore, directions of the AB spindles were assumed to deviate, on average, by 36° from the A-P axis as described previously [36]. Calculation of the apical and basolateral axes and the exposed surfaces of E and MS were performed from fluorescent micrographs in ImageJ.

### Laser ablations

For ablation experiments, a dye laser was used, and ablation was controlled through a tunable dye laser system (MicroPoint; Photonic Instruments Inc., St. Charles, IL, USA) attached to the microscope, which allows simultaneous observation and irradiation of cells. Settings were: dye cell filled with 5 mmol/l Coumarin 440 (peak at 435 nm), 10 to 20 pulses (duration of 0.6 to 1.2 seconds), 5 repetitions, attenuator plate at position 4 to 6 or 50%. Larger early blastomeres were irradiated sequentially during a time interval for up to 5 minutes in the area of the nucleus. The degree of ablation was judged from the bleaching of the GFP::HIS-72 nuclear marker. Blastomeres irradiated in this way were not completely arrested, and usually produced later aberrant divisions. Ablations of the Ea cortex were performed essentially as detailed above, but with, GFP::NMY-2 used as ablation marker and ablations carried out in 3 to 4 pulse sequences applied in 1 minute.

### Visualization of morphogenetic cell movements

Visualizations were generated using custom programs generated in MATLAB software (MathWorks), which will be published elsewhere. Briefly, to calculate migration paths, we used embryos lineaged with StarryNite (http://starrynite.sourceforge.net) and AceTree software [57], and extracted each cell's position in three dimensions, one along each developmental axis. Correlation coefficients were calculated for pairs of cells based on their movement along the left/right axis throughout the time they were both present in the embryo. These values ranged between -1 and 1 m and color values are displayed for the range between -0.8 and 0.8. Furthermore, migration paths were smoothed using a moving average with a width of five time points.

### Three-dimensional reconstructions

Cell shapes were reconstructed from three-dimensional (3D) time-lapse microscopy images by manually tracing the outline of cells with a 3D editing plug-in for ImageJ (UCLA, Los Angeles, CA, USA [60]). The output is a. shapes file that contains the outlines in each two-dimensional confocal plane. These outlines were recalculated with a custom MATLAB script and imported into DAZ software [61] for further rendering.

## Competing interests

The authors declare that they have no competing interests.

## Authors' contributions

CP conceived, designed, and carried out the experiments. CP and ZB analyzed the data and wrote the manuscript. MT contributed to the generation of strains and time-lapse recordings. AS and JLM contributed to the computational analysis. All authors read and approved the final version of the manuscript.

## Supplementary Material

Additional file 1 **Cell internalization during gastrulation, ventral surface**. The movie starts when the endoderm begins to internalize. Internalizing cells and cell groups forming rosettes are outlined in white. The movie briefly stops to indicate the respective morphogenetic event. Scale bar = 10 μm, frame rate is 4.5 min/s. Click here for file

Additional file 2 **Endoderm internalization**. Animation of three-dimensional (3D) reconstructions of cell shapes during endoderm internalization. Click here for file

Additional file 3 **Analyses of Ea/Ep internalization and apical dynamics. (A) **Morphometric analysis of apical cell shape. (Top left) Drawing depicting the directions in which the measurements were performed. Flatness was calculated using the depicted formula 20]. Shapes were measured from two-dimensional (2D) confocal images (mean ± SD, n = 3). **(B) **Ea/Ep are arc-shaped during internalization. Ventral (blue), sagittal (green)m and transverse (yellow) views of the Ea/Ep apical surfaces generated by projecting three-dimensional (3D) volume data in the respective direction. **(C) **Flows of contractile foci on Ea shortly after cell birth and during internalization. Superimposition of five consecutive frames from cortical projection stills recorded at 2-second intervals. Streams of non-muscle myosin (NMY)-2 foci are highlighted by arrowheads. **(D) **Kymographs of contractile flows on Ea starting at time points corresponding to (A), and generated along a line similar to the white dashed line in Figure 1A. Arrows indicate local contractile flow convergence that led to extension advancement. **(E) **Transient covering of cells by lateral extensions. (Top) Cortical views of ABplapp. Dashed lines outline the extensions of surrounding cells, and progression of covering and resurfacing is shown in the bottom right frame. (Bottom) Quantification of the degree of covering over time (mean ± SD, n = 3). **(F) **Apical dynamics of Partitioning defective (PAR)-6 (representations as in Figure 1A). White arrows mark the accumulation of PAR-6 on the apical surface of Ea, red arrow marks the accumulation on ABalppp, which later internalized later (see Figure B). White lines denote sister cells. **(G) **Repolarization of Ea/Ep during internalization. (Left) Side view of Ea with average projection of 2 focal planes at 1 um apart in the middle of the embryo. (Right) Quantification of PAR-2::GFP along the perimeter of Ea (inside the white dashed lines) in counterclockwise direction from the gray arrow to the black arrow. Click here for file

Additional file 4 **Covering depended on surrounding cells**. Irradiation of**(A) **P_3 _or **(B) **other neighboring AB cells prevented Ea/Ep internalization. (Top panels) Time-lapse microscopy with three-dimensional (3D) projection still images. Lightning marks the irradiated cells. Mesoderm is outlined with dashed lines, non-internalized endoderm is marked with a white circle, and arrow points to the center of the rosette. (Bottom panels) Schematic illustration depicting the embryo in the same orientation as above, and highlighting the irradiated cell. Click here for file

Additional file 5 **Cellular dynamics during rosette formation and its developmental plasticity. (A) **Cortex rupture and bleb formation in Ea/Ep. (Top) Side view of two-dimensional (2D) confocal slices. White arrows point to breakpoints in the cortex, while the red arrow marks a reemerging NMY-2 focus at the tip of the bleb. (Bottom left) Schematic representation of blebbing events in a representative embryo. Blebs are indicated by arrows, and the dashed white line indicates the initial position of Ea/Ep apices. (Bottom right) Size distribution of blebs shown on the left. **(B) **Effect of non-muscle myosin (NMY)-2 RNA interference (RNAi) on blebbing and internalization dynamics. (Top) Cortical views overlaying cellular membranes (gray) with color-coded NMY-2 intensities. (Right) Covering kinetics and number of blebs in wild-type and *nmy-2 *RNAi-treated embryos. **(C) **Cells in rosettes divided along the long axis. (First panel) three-dimensional (3D)projection image shows polarization of cells in rosettes. (Second panel) Quantification of long and short axis of cells from 3D still images (mean ± SD, n = 5). (Third panel) Cell polarization during rosette formation. (Fourth panel) Division orientation in the same embryos as in third panel (double arrows) (n = 5). **(D) **Cellular topology during endoderm internalization in three different nematodes. Color code for tissues is indicated on the bottom left, and direction of covering with black arrows. Click here for file
