# Supplementary figures and images for: Actomyosin-based Self-organization of cell internalization during C. elegans gastrulation
Source: BMC Biol. 2012 Nov 30;10:94. doi: 10.1186/1741-7007-10-94 (PMC3583717; doi:10.1186/1741-7007-10-94)

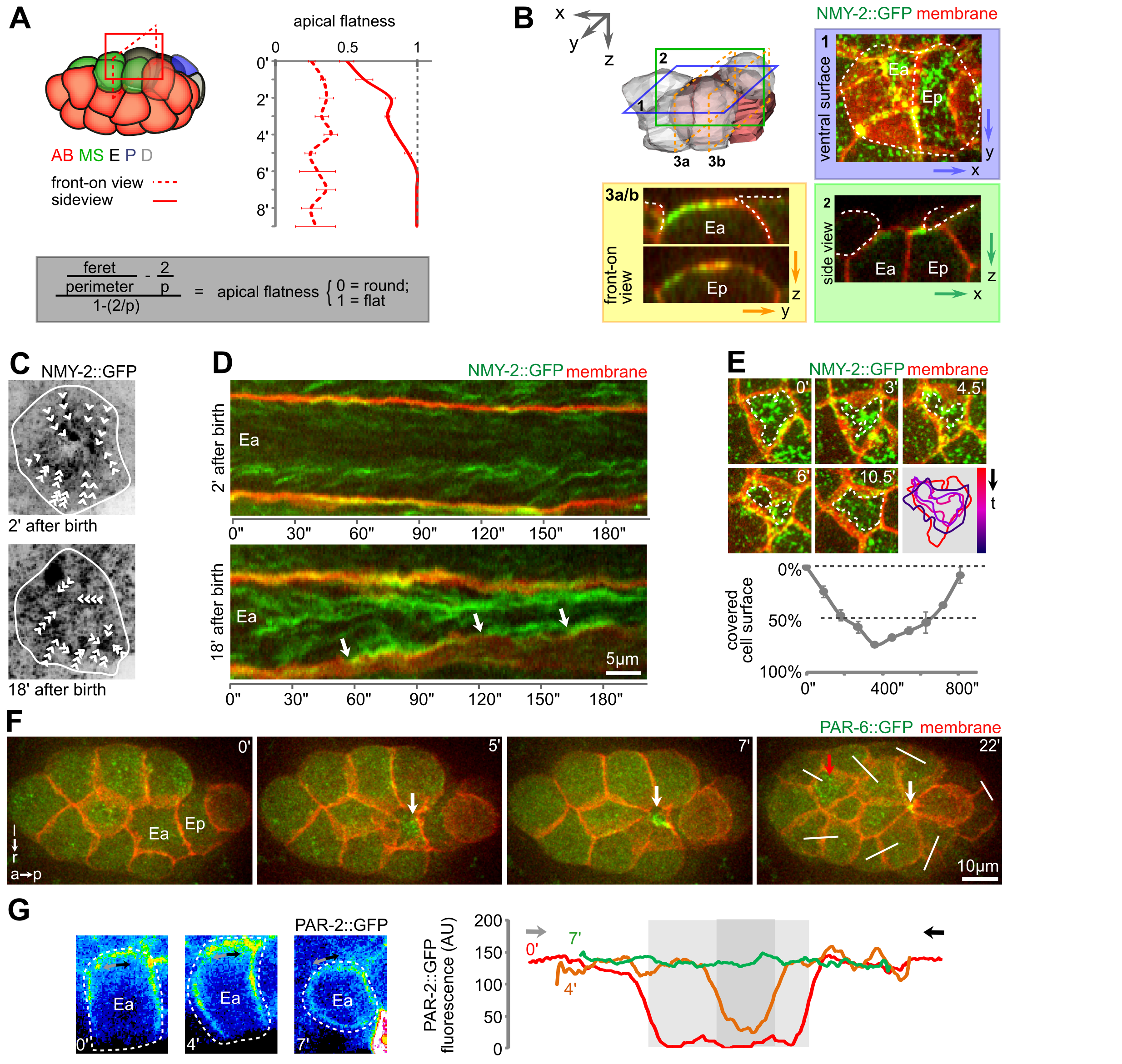

Supplement: Additional file 3 — Analyses of Ea/Ep internalization and apical dynamics. (A) Morphometric analysis of apical cell shape. (Top left) Drawing depicting the directions in which the measurements were performed. Flatness was calculated using the depicted formula 20]. Shapes were measured from two-dimensional (2D) confocal images (mean ± SD, n = 3). (B) Ea/Ep are arc-shaped during internalization. Ventral (blue), sagittal (green)m and transverse (yellow) views of the Ea/Ep apical surfaces generated by projecting three-dimensional (3D) volume data in the respective direction. (C) Flows of contractile foci on Ea shortly after cell birth and during internalization. Superimposition of five consecutive frames from cortical projection stills recorded at 2-second intervals. Streams of non-muscle myosin (NMY)-2 foci are highlighted by arrowheads. (D) Kymographs of contractile flows on Ea starting at time points corresponding to (A), and generated along a line similar to the white dashed line in Figure 1A. Arrows indicate local contractile flow convergence that led to extension advancement. (E) Transient covering of cells by lateral extensions. (Top) Cortical views of ABplapp. Dashed lines outline the extensions of surrounding cells, and progression of covering and resurfacing is shown in the bottom right frame. (Bottom) Quantification of the degree of covering over time (mean ± SD, n = 3). (F) Apical dynamics of Partitioning defective (PAR)-6 (representations as in Figure 1A). White arrows mark the accumulation of PAR-6 on the apical surface of Ea, red arrow marks the accumulation on ABalppp, which later internalized later (see Figure B). White lines denote sister cells. (G) Repolarization of Ea/Ep during internalization. (Left) Side view of Ea with average projection of 2 focal planes at 1 um apart in the middle of the embryo. (Right) Quantification of PAR-2::GFP along the perimeter of Ea (inside the white dashed lines) in counterclockwise direction from the gray arrow to the black arrow. [file 1741-7007-10-94-S3.PNG]

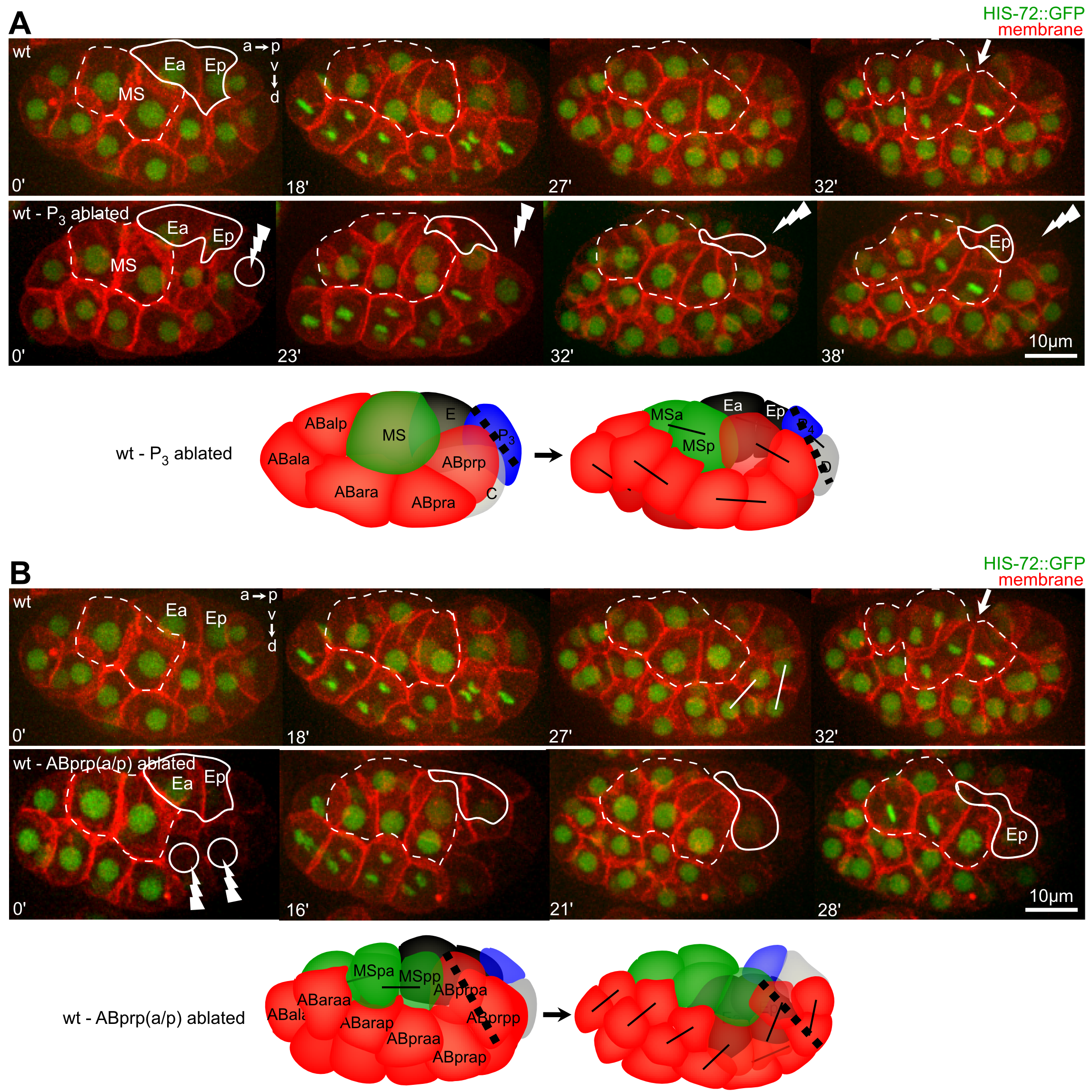

Supplement: Additional file 4 — Covering depended on surrounding cells. Irradiation of(A) P3 or (B) other neighboring AB cells prevented Ea/Ep internalization. (Top panels) Time-lapse microscopy with three-dimensional (3D) projection still images. Lightning marks the irradiated cells. Mesoderm is outlined with dashed lines, non-internalized endoderm is marked with a white circle, and arrow points to the center of the rosette. (Bottom panels) Schematic illustration depicting the embryo in the same orientation as above, and highlighting the irradiated cell. [file 1741-7007-10-94-S4.PNG]

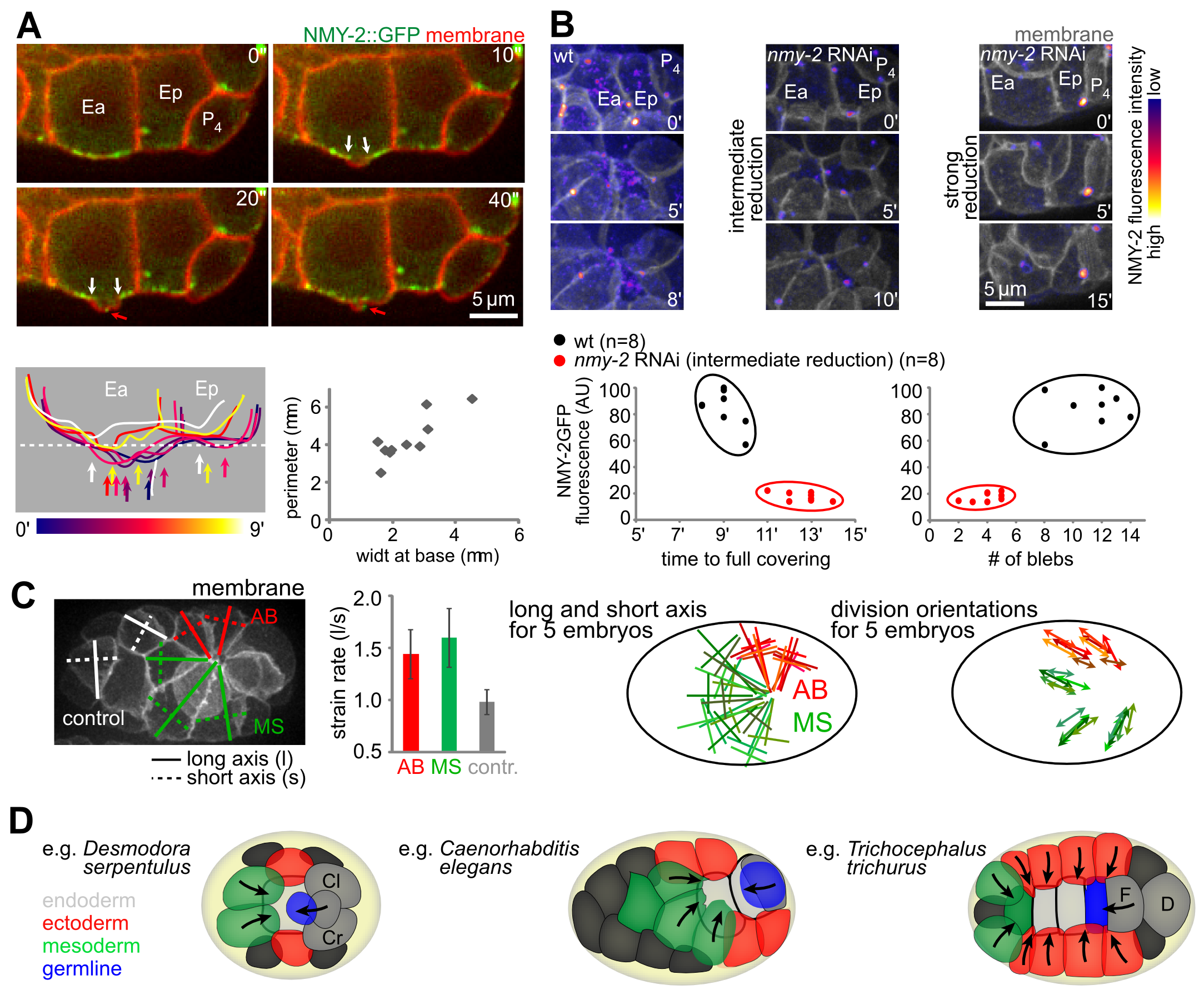

Supplement: Additional file 5 — Cellular dynamics during rosette formation and its developmental plasticity. (A) Cortex rupture and bleb formation in Ea/Ep. (Top) Side view of two-dimensional (2D) confocal slices. White arrows point to breakpoints in the cortex, while the red arrow marks a reemerging NMY-2 focus at the tip of the bleb. (Bottom left) Schematic representation of blebbing events in a representative embryo. Blebs are indicated by arrows, and the dashed white line indicates the initial position of Ea/Ep apices. (Bottom right) Size distribution of blebs shown on the left. (B) Effect of non-muscle myosin (NMY)-2 RNA interference (RNAi) on blebbing and internalization dynamics. (Top) Cortical views overlaying cellular membranes (gray) with color-coded NMY-2 intensities. (Right) Covering kinetics and number of blebs in wild-type and nmy-2 RNAi-treated embryos. (C) Cells in rosettes divided along the long axis. (First panel) three-dimensional (3D)projection image shows polarization of cells in rosettes. (Second panel) Quantification of long and short axis of cells from 3D still images (mean ± SD, n = 5). (Third panel) Cell polarization during rosette formation. (Fourth panel) Division orientation in the same embryos as in third panel (double arrows) (n = 5). (D) Cellular topology during endoderm internalization in three different nematodes. Color code for tissues is indicated on the bottom left, and direction of covering with black arrows. [file 1741-7007-10-94-S5.PNG]
